# Data-Driven Chance-Constrained Mixed Integer Nonlinear Bi-level Optimisation Via Copulas: Application To Integrated Planning And Scheduling Problems

**DOI:** 10.69997/sct.169891

**Published:** 2025-06-27

**Authors:** Syu-Ning Johnn, Hasan Nikkhah, Meng-Lin Tsai, Styliani Avraamidou, Burcu Beykal, Vassilis M. Charitopoulos

**Affiliations:** a University College London, Department of Chemical Engineering, The Sargent Centre for Process Systems Engineering, London, WC1E 7JE, UK; b University of Connecticut, Department of Chemical & Biomolecular Engineering, Storrs, CT, USA; c University of Connecticut, Center for Clean Energy Engineering, Storrs, CT, USA; d University of Wisconsin-Madison, Department of Chemical & Biological Engineering, Madison, WI, USA

**Keywords:** Planning & Scheduling, Derivative Free Optimization, Bi-level Optimization, Copula Theory, Data-driven optimization

## Abstract

Planning and scheduling are integral components of process supply chains. The presence of data correlation, particularly multivariate demand data dependency, can pose significant challenges to the decision-making process. This necessitates the consideration of dependency structures inherent in the underlying data to generate good-quality, feasible solutions to optimisation problems such as planning and scheduling. This work proposes a chance-constrained optimisation framework integrated with copulas, a non-parametric data estimation technique to forecast uncertain demand levels in accordance with specified risk thresholds in the context of a planning and scheduling problem. We focus on the integrated planning and scheduling problem following a bi-level optimisation formulation. The estimated demand forecasts are subsequently utilised within the Data-driven Optimisation of bi-level Mixed-Integer NOnlinear problems (DOMINO) framework to solve the integrated optimisation problem, and derive decisions with guaranteed demand satisfaction rates. Computational experiments demonstrate that our proposed copula-based chance-constrained optimisation framework can incorporate demand correlation and achieve higher joint demand satisfaction rate, lower total costs with higher efficiency.

## INTRODUCTION

In real-world applications, many optimisation problems are inherently difficult to find feasible solutions due to the lack of exact information, the presence of noisy data distributions, data correlations, and parameter uncertainties. As a result, data-driven optimisation approaches are increasingly adopted to efficiently explore solution spaces and identify improved outcomes [2]. Chance-constrained programming (CCP) is an optimisation-based approach that ensures stochastic constraints are met with a predetermined probability of satisfaction amongst all possible scenarios [5, 17]. Numerous studies have successfully integrated CCP within various optimisation problems [3]. However, the applicability of CCP to general optimisation problems is limited by the need of strict assumptions regarding the stochastic nature of the parameters studied. To this end, data-driven variants of CCP have gained increasing attention [15]. Copulas are data-driven coupling functions that capture the dependence structure between multiple uncertain parameters with inherent correlations. Incorporating copula formulations into CCP has the advantage of better modelling dependencies between variables under different scenarios when the underlying data exhibits complex distributions or non-trivial dependencies, such as correlated risks, thereby improving the accuracy of decision-making in optimisation problems with the presence of uncertain parameters. In recent years, the integration of copula and CCP has shown significant promise. [16] formulated/employed joint chance constraints to address the capacity requirement in a multi-commodity network flow problem, using an Archimedean copula to estimate the dependencies between different capacity coefficient variables. [14] used a copula to model the dependency between resource consumptions of a stochastic resource-constrained shortest path problem. [11] proposed a joint chance-constrained method that integrates the best-performing copula from a set of widely applied bivariate copulas to characterise the uncertain probability distributions and the correlated nature of multi-period, multi-zone residential waste data.

In this work, we propose a data-driven chance-constrained optimisation framework to estimate the demand level within the context of planning and scheduling by leveraging the capabilities of copulas, which helps to achieve good efficiency and accuracy in estimating the multivariate demand data dependency structure. Moreover, we integrate our proposed framework with the DOMINO (Data-driven Optimisation of bi-level Mixed-Integer NOlinear problems) framework [2], which is a data-driven grey-box algorithm for addressing bi-level optimisation problems with various formulations at the lower levels [1].

## METHODOLOGY

### Copula Theory

A copula is a function that joins or “couples” multiple univariate marginal distributions to form a multivariate distribution. This enables the construction of any multivariate distribution using specified marginal distributions and the chosen copula, regardless of whether the underlying real distribution is fully understood. At a higher level, copulas are essential statistical tools for comprehensively analysing the dependency structures that exhibit in multiple random variables, effectively bypassing the marginal behaviour from joint dependence. By focusing on the joint cumulative distribution of marginal quantiles, copulas can capture complex, non-linear, and tail dependencies (extreme events) that traditional correlation metrics may often overlook [4].

The mathematical foundation of a copula is derived from Sklar’s Theorem, which states that for any *n*-dimensional cumulative distribution function *F* with marginal distributions *F*_1,_ *F*_2,_ … *F*_*n*_, there exists a copula *C* that models and encapsulates the dependency structure:

(1) $$F\left(x_{1},x_{2},\ldots x_{n}\right)=C\left(F_{1}\left(x_{1}\right),F_{2}\left(x_{2}\right),\ldots F_{n}\left(x_{n}\right)\right)$$

where *x*_1,_ *x*_2,_ … *x*_*n*_ are realisations of the random variables from each distribution, and the marginal distributions *F*_*i*_(*x*_*i*_), *i* ∈ {1, … *n*} represent the individual variable behaviour. A few commonly applied copula models include Gaussian copula [18], t-copula [7], and Archimedean copula [13], each tailored to specific types of dependency structures.

### Chance constrained programming

Chance-constrained programming (CCP) is an optimisation technique that formulates probabilistic constraints to model uncertainty in decision-making problems [17]. This approach addresses uncertainty by allowing specific constraint(s) to be satisfied with a predetermined probability level, thus offering controlled flexibility to manage risk and account for the inherent variabilities in decisions related to demand, resource, or time availability. As a result, CCP provides a structured way to accommodate unpredictable conditions for many real-world problems in fields like energy production [23], finance [6], and supply chain management [19].

An individual probabilistic constraint, known as an individual chance constraint (ICC), requires that the constraint be met with a certain confidence level, denoted as a probability term (1 – *α*)%, where *α* represents the risk level [5]. For example, setting *α* = 0.05 means that the constraint must hold with at least 95% probability while allowing for a 5% risk tolerance for potential constraint violation. The formulation of a basic ICC can be represented as follows in the context of a production problem.

(2) $$P\left[x_{i}\geq D_{i}\right]\geq 1-\alpha_{i},i\in \{1,\ldots ,n\}$$

where for each production constraint *i*, the production level decision variable *x*_*i*_ must fulfil the uncertain demand parameter *D*_*i*_ for at least (1 – *α*_*i*_)% of the time. Joint chance constraint (JCC) is a variation of ICC, stressing that multiple constraints must be satisfied simultaneously with a given confidence level. Similarly, the JCC formulation can be expressed mathematically as:

(3) $$P\left[x_{i}\geq D_{i},i\in \{1,\ldots ,n\}\right]\geq 1-\alpha$$

where the production level *x*_*i*_ in all production scenarios *n* must be sufficient to meet uncertain demand collectively with a joint confidence level of (1 – *α*)% As a result, JCCs impose stricter limitations on the feasible region of the solution space, making the problem generally more complex to solve.

The cumulative distribution function (CDF) provides a foundational basis for CCP in accumulating probabilities and facilitates the probabilistic guarantee of constraint satisfaction up to a given threshold. On the contrary, the quantile function, calculated as the inversed CDF, pinpoints a target probability level to the corresponding minimum production value required to meet the specified probability threshold.

### Data-driven chance constrained programming via copulas

In real-life applications, formulating chance constraints is generally challenging because the probability distribution of the uncertain parameters is often unknown or difficult to model with limited observed data. The difficulty of estimating an unknown distribution by existing parametric distributions (e.g., Gaussian distribution) is pronounced in high-dimensional or nonlinear contexts with no closed-form formulations, which often necessitates approximation or data-driven methods to adequately capture the distribution. This challenge becomes even more complex with JCCs, in which the dependency structure among the marginal demand distributions introduces correlation in the data. To tackle this issue, integrating the copulas technique into the modelling of JCCs offers a data-driven approach based on empirical data that models the marginal distributions of uncertain variables and their dependency structure to construct joint distributions.

**Figure 1.**
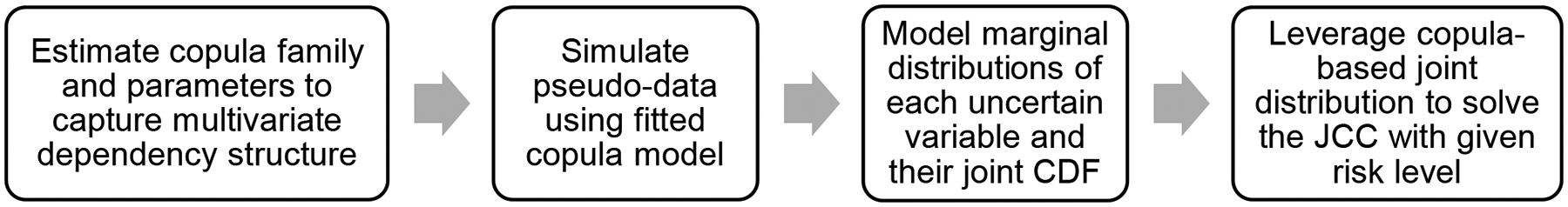
Logic flow for the proposed data-driven JCC framework with copula.

We propose a data-driven framework that integrates copulas into data-driven JCC optimisation, as illustrated in Figure 1. We first employ pyvinecopulib [20] to fit the copulas to existing demand data using maximum likelihood estimation to estimate the best-fitting dependency structure and determine the copula family as well as its parameters. The selection of specific copulas family and dependency structure are based on optimising the AIC, BIC and log likelihood statistics using the pyvinecopulib package. Then, we use the fitted copula to construct a surrogate model that simulates correlated pseudo data while preserving the same dependency structure. The simulated pseudo data offers the advantage of scalability and enhancement by generating complete datasets with dependency structure when the original data may contain real-world data biases or missing values. Then, we can estimate the marginal distributions via non-parametric methods such as kernel density estimation [5] and in this approach, we adapt the built-in function in pyvinecopulib to estimate the marginal distributions and the joint CDF. Lastly, once the copula and marginal distributions are defined, the joint distribution can be estimated via (1) and utilised for approximating the quantile function and the risk level.

Following the data-driven copula framework above, we evaluate the JCC using Monte Carlo simulation. Each scenario generated from the copula-based quantile function is employed to compute the probability of constraint satisfaction across all realisations. By adjusting the number of empirical simulations, the confidence level for the JCCs can be calibrated to meet the desired level of risk tolerance *α*.

### Data-driven mixed integer nonlinear bi-level optimisation

**Figure 2.**
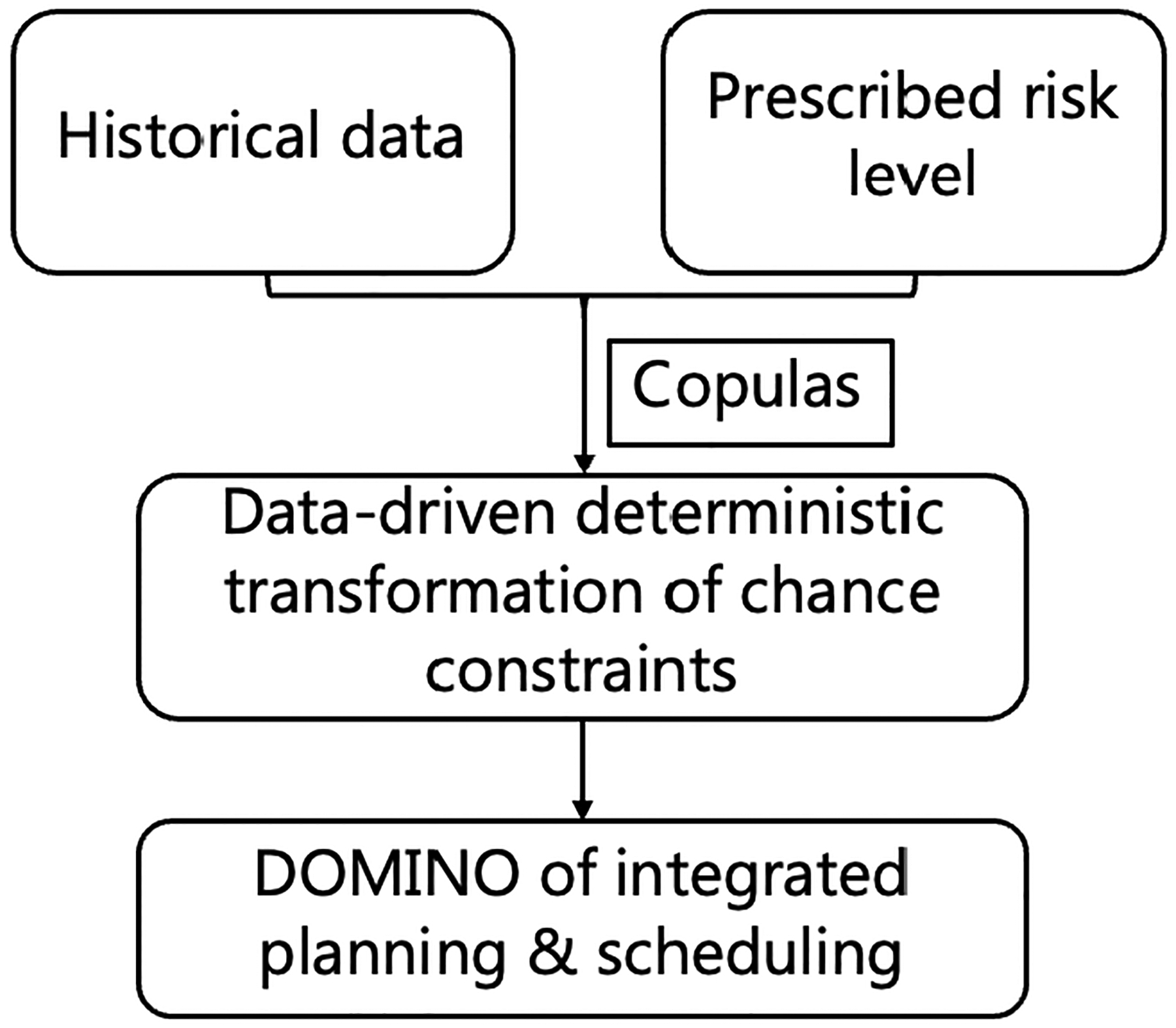
Integrated copulas-DOMINO solution workflow

Historical production demand data of an integrated planning and scheduling problem is utilised to compute the copula-estimated demand set corresponding to a particular risk level following the procedure shown in Figure 1. The generated demand set can then be utilised as a demand scenario of integrated planning and scheduling optimisation problems [8, 10, 12] to be solved using the DOMINO framework. DOMINO is a data-driven strategy for solving general constrained bi-level optimisation problems which transform bi-level problems into single-level grey-box optimisations, utilising advanced data-driven techniques and deterministic solvers [22]. We refer the interested reader to [2] for a detailed description of the DOMINO framework and its application to solve integrated planning and scheduling problems under demand uncertainty in [1]. Figure 2 illustrates the workflow between the copulas and DOMINO framework. CCP offers a simplified uncertainty estimation approach based on risk level, which is more efficient compared to alternative methods such as stochastic programming, as originally proposed in [1], which requires explicit representation of all scenarios and since an increasing number of scenarios gives us a better understanding, incurs computational burden for high-dimensional problems.

## CASE STUDY

### Experimental setup

We employ our proposed approach to tackle the integrated planning and scheduling problem under product demand uncertainty. We consider a continuous single stage process with 2 products and 7 days, each with a 24hr duration. Firstly, we define the desired risk level and generate the corresponding demands, which are input as production targets into the DOMINO framework to tackle data-driven optimisation of the production level and evaluate the demand satisfaction rate via a simulation-based evaluation process. Throughout the experiment, two independent sets of scenario-based demands are generated: the first set is utilised to train the copula model designed to predict the empirical CDF and determine the quantile function for the joint chance constraint on demands. The second set serves as an evaluation benchmark during the simulation process to compute the demand satisfaction rate.

We employ the DOMINO framework for the solution of a bi-level optimisation problem case study with linear production planning and mixed-integer nonlinear scheduling from [21], and linear programming planning and scheduling case study from [1], respectively. Specifically, the nonlinear aspects of the integrated problem stem from the variable transition times in the scheduling of continuous manufacturing processes [9]. The case study is executed 10 times on a High-Performance Computing (HPC) machine using Red Hat Enterprise Linux 8.9 (Ootpa). The NOMAD algorithm within the DOMINO framework is executed serially using 1 core per node with 48GB RAM, starting from a random initial point.

### Results

**Figure 3.**
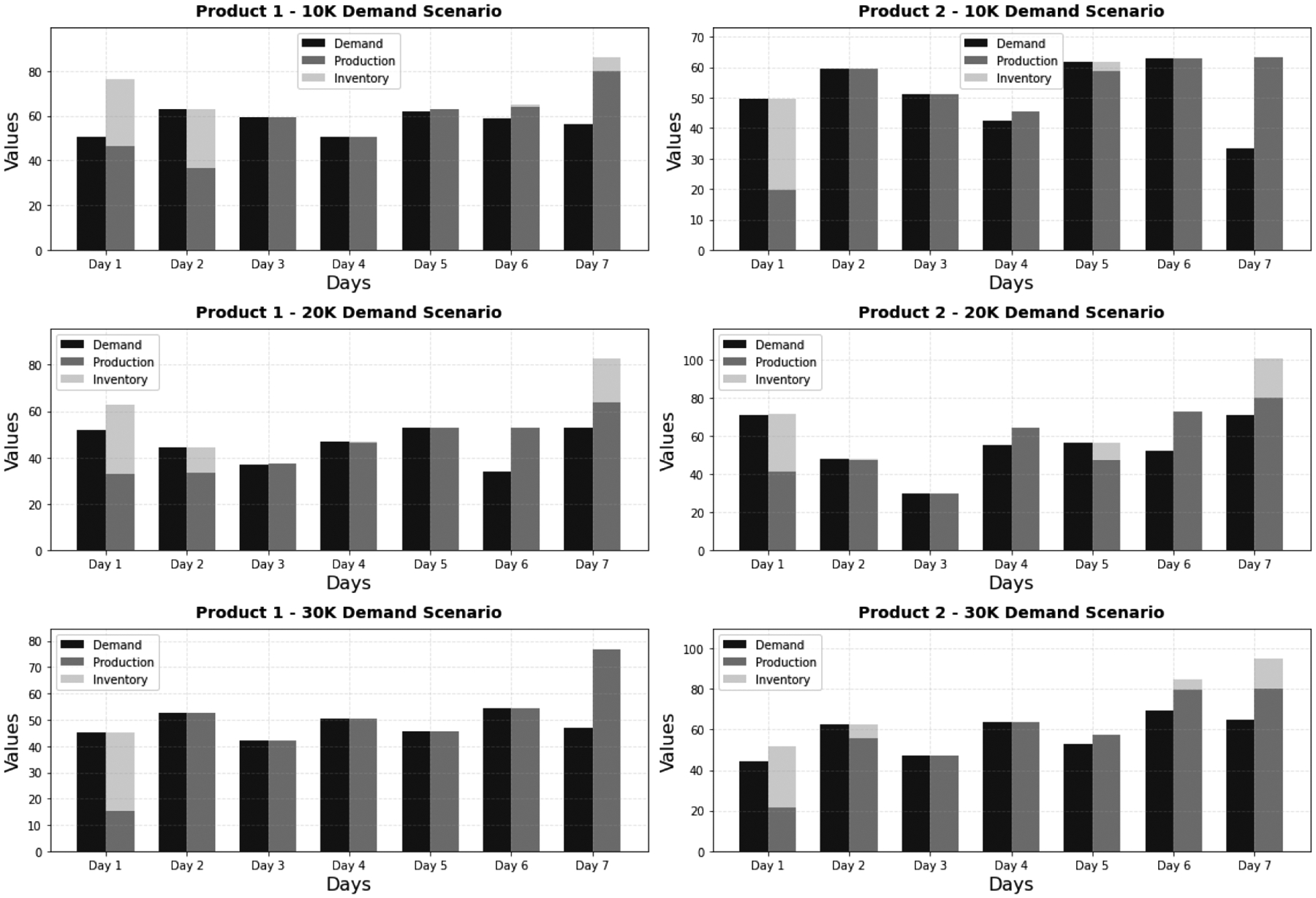
Production and inventory levels for the 2-product 7-day instance with copula-generated demand level under a specific number of scenarios (10, 20 or 30K). Planning cost values (scenario sizes): $7,085 (10K), $7,009.5 (20K), $6,850 (30K).

We compute the copula-estimated demand set and input it into the DOMINO framework, wherein the integrated planning and scheduling problem is addressed. The production and inventory level outcomes are presented in Figure 3.

In our experimental design, we aim for 99% demand satisfaction during the generated production schedules. To evaluate, we will assess how the actual performance (a posteriori) in a real-world environment compares to our initially established reliability level (a priori) based on the copula-generated production target. That is, we calculate a fixed schedule derived from the copula-estimated demand level and aim to fix this target as a baseline. Subsequently, we employ copula to simulate the demand and evaluate the frequency with which the simulated demand satisfies all predefined scenarios.

The discrepancy between the a priori and a posteriori performance can arise because the copula method is data-driven and non-parametric. Since we do not require any prior assumption about the underlying distribution for the demand data, we rely on the copula model to estimate the CDF of joint and individual demands. To analyse the impact of this discrepancy, we conduct the following experiment to evaluate the extent to which the scenario size employed during the copula model training influences the accuracy of prediction regarding demand satisfaction. Specifically, we utilise the joint and individual demand satisfaction rates as metrics to assess the quality of copula-generated demand under a certain risk level. The joint satisfaction rate represents the proportion of simulated scenarios for which all product demand targets are met. In contrast, the marginal satisfaction rate for each demand is individually independent, and the multiplication of these rates gives the independently joint satisfaction level, assuming all demands are individually met without considering any intercorrelation.

Table 1 showcases the satisfaction rate and computational time required to solve the offline copula model for different instance and scenario sizes with 7 days. Results demonstrate that the copula model, which assumes correlation among demands, yields higher joint satisfaction rates than the multiplication of individual rates, highlighting the copula model’s capacity to capture the collective behaviour of demand data. Moreover, a higher joint satisfaction rate produced by copula suggests that interactions between demand levels may confer additional value. Incorporating these interactions could be crucial in increasing the overall demand satisfaction rate among all products. Furthermore, Table 1 shows a monotonic increase in the joint satisfaction rate when the total number of training scenarios increases from 10k to 30k for all instance sizes. This suggests that increasing the number of scenarios used to train the copulas model can enhance the accuracy of the estimated CDF, resulting in generating a fixed production level that ultimately raises the likelihood of demand satisfaction. The average computational time required for DOMINO to solve the integrated planning and scheduling problem is approximately 6 hours for the case study of 2 products and 7 days. This underscores the significance of managing uncertainty in an offline manner, as advocated by our proposed data-driven copulas framework. Additionally, our proposed copula approach requires estimating the CDF and solving the model only once, hence further enhancing its efficiency.

To summarise, we notice the advantages of our copula method are that the copula-generated plan (as an input to DOMINO) will be feasible for every possible scenario realisation for 99% of the times. Moreover, instead of solving the scenarios iteratively (as in [1]) and being very time-consuming, we can compute the copula-estimated CDF offline to massively save time at the cost of being slightly conservative (risk averse) with the actual demand satisfaction.

### Concluding remarks

We present a joint chance-constrained optimisation framework with data-driven copulas functions to effectively capture and model the multivariate demand data dependency structure for an integrated planning and scheduling problem. Our approach ensures feasible decision-making within a defined risk threshold. We validated the data-driven copulas-generated production targets using the DOMINO framework and tackled the data-driven optimisation. Our experiments demonstrate that the proposed approach is capable of identifying robust solutions that result in higher joint satisfaction rates for products and near-optimal performance, all while significantly reducing computational time compared to exact or stochastic optimisation methods. Plans for future research involve validating the efficiency and effectiveness of our approach through a number of case studies across an extended range of optimisation problems.

## Figures and Tables

**Table 1:** Computational time and satisfaction rates for scenario sizes (averaged of 5 computational rounds).

Products	Weeks	Scenarios	Compute Time (sec)	Multiplication of Individual Satisfaction Rates	Copula-estimated Joint Satisfaction Rates
1	1	10k	78	99.4%	100%
1	1	20k	255	98.8%	100%
1	1	30k	486	99.1%	100%
2	1	10k	81	98.5%	99.9%
2	1	20k	234	98.3%	100%
2	1	30k	585	98.1%	100%
4	1	10k	109	96.4%	98.6%
4	1	20k	281	96.2%	99.1%
4	1	30k	527	96.3%	99.3%
6	1	10k	372	93.1%	96.4%
6	1	20k	516	94.0%	96.8%
6	1	30k	842	93.8%	97.0%
